# ICP-MS based seasonal and spatiotemporal evaluation of potentially toxic and major elements in surface waters of Akdağ National Park, Türkiye

**DOI:** 10.1038/s41598-026-35053-z

**Published:** 2026-02-13

**Authors:** Zeyneb Karakuş, Recep Kara, Mustafa Yalçın, Hakan Yılmaz, Hesna Kandır, Abdurrahman Fatih Fidan

**Affiliations:** 1 Department of Chemistry and Chemical Processing Technologies, Çay Vocational School, Afyon Kocatepe University, Afyonkarahisar, Türkiye; 2 Department of Food Hygiene and Technology, Faculty of Veterinary Medicine, Afyon Kocatepe University, Afyonkarahisar, Türkiye; 3 Department of Geomatics Engineering, Faculty of Engineering, Afyon Kocatepe University, Afyonkarahisar, Türkiye; 4 Department of Cinema and Television, Faculty of Fine Arts, Afyon Kocatepe University, Afyonkarahisar, Türkiye; 5 Department of Wild Animal Diseases and Ecology, Faculty of Veterinary Medicine, Afyon Kocatepe University, Afyonkarahisar, Türkiye; 6 Department of Biochemistry, Faculty of Veterinary Medicine, Afyon Kocatepe University, Afyonkarahisar, Türkiye

**Keywords:** ICP-MS, GIS mapping, Spatial interpolation, Freshwater quality, PTMEs, Akdağ national park, Ecology, Ecology, Environmental sciences, Hydrology, Water resources

## Abstract

Understanding how potentially toxic and major elements (PTMEs) vary in freshwater systems is essential for evaluating environmental and public health risks. This study explored the seasonal and spatial variations of PTMEs in the surface waters of Akdağ National Park, Türkiye, using inductively coupled plasma-mass spectrometry (ICP-MS).Water samples were collected from nine stations across four seasons and analyzed using inductively coupled plasma-mass spectrometry (ICP-MS). Among the trace elements, Fe, Mn, and Al ranged from 120-460 µg/L, 15-310 µg/L, and 70-380 µg/L, respectively, while Pb and As remained low (1.2-8.4 µg/L and 2.3-6.1 µg/L), all within international safety limits. In contrast, Ca and Mg concentrations (18-72 mg/L and 3.5-12 mg/L) mainly reflected local geological conditions rather than contamination. Spatial interpolation (IDW) within a GIS framework indicated slightly higher PTME levels at certain locations compared with less affected sites. Overall, the results provide the first spatiotemporal baseline of PTMEs for this protected area, emphasizing the need for continuous monitoring to support sustainable water resource management and ecosystem conservation.

**Supplementary Information:**

The online version contains supplementary material available at 10.1038/s41598-026-35053-z.

## Introduction

Water is an essential component for the survival of all living organisms and is one of the most widespread natural resources on Earth. Approximately 75% of the Earth’s surface is covered by water. However, 97% of this water is found in oceans and seas, 2% is found in groundwater, lakes, and rivers, and only 1% is found in glaciers and the atmosphere. Despite the abundance water in general, usable freshwater resources constitute only about 1% of the total water resources on Earth, posing a significant limitation for drinking water supply and ecosystem sustainability.

Today, rapid industrialization, agricultural activities, and urbanization have led to increasing threats to water resources in terms of both quality and quantity. Pollutants continuously discharged into the natural environment re-enter the water cycle, adversely affecting the physical, chemical, and biological characteristics of aquatic ecosystems^1^. The direct or indirect discharge of industrial, agricultural, and domestic waste into water bodies severely threatens the quality of both surface water and groundwater, with serious implications for ecosystem and public health.

In this context, according to the Surface Water Quality Management Regulation published in the Official Gazette dated 15.04.2015 and numbered 29327 in Türkiye, water pollution is defined as the negative alteration of the chemical, physical, bacteriological, radiological, and ecological characteristics of water resources. Water pollution is recognized as a major global environmental problem and represents a significant risk for Türkiye.

In particular, lake and river ecosystems are increasingly threatened by eutrophication, toxic chemical pollution, thermal pollution, and various anthropogenic pressures^2^. One of the most critical pollutant groups in water resources is PTMEs. These elements, which are difficult to degrade biologically and have a tendency to accumulate in the environment, can enter the food chain once they accumulate in aquatic ecosystems, causing long-term ecotoxicological effects^3,4^. Although PTMEs naturally occur in trace amounts in water resources, industrial and agricultural activities can elevate them to toxic levels, posing a potential threat to aquatic organisms^5^. PTMEs in dissolved form can be absorbed by aquatic organisms, accumulate in their tissues and organs, and cause serious biological hazards such as oxidative stress, protein denaturation, and DNA damage^6^.

Recent advancements in geospatial technologies have significantly enhanced the monitoring and evaluation of water quality, especially through the integration of Geographic Information Systems (GIS) and spatial interpolation techniques such as Inverse Distance Weighting (IDW). Oliveira et al.^7^ conducted a comprehensive assessment of surface water quality in three agricultural micro-watersheds in southern Brazil by GIS and applied the IDW method to map spatial variations in pollution levels. Similarly, Paiva et al.^8^ employed coupled hydrological and water quality models within a GIS framework to simulate parameter distributions in paired watersheds of the Doce River, emphasizing the impacts of land use and domestic effluent discharge on spatial water quality patterns. In a different context, Hossain et al.^9^ investigated groundwater contamination in coastal Bangladesh using a multi-indexing approach within a GIS environment, where spatial interpolation techniques facilitated the visualization of contaminant distributions and associated ecological and health risks. These studies have collectively highlighted the efficacy of GIS-based analysis and IDW interpolation in identifying pollution hotspots, supporting risk assessment, and guiding sustainable water resource management in diverse environmental settings. Islam et al.^10^ presented a refined approach to region-specific water resource management in a GIS environment by using the IDW method in conjunction with multivariate statistical analysis to assess seasonal and spatial variations in irrigation water quality in the Old Brahmaputra River in Bangladesh. Similarly, Halder^11^ employed a GIS-based analysis to assess the suitability of groundwater for both drinking and irrigation in Gangetic West Bengal, revealing the spatial extent of quality variations linked to hydrogeochemical processes. In Kuwait, Al-Ruwaih et al.^12^ applied GIS techniques alongside water quality index assessments to determine the agricultural and industrial applicability of groundwater from the Dammam aquifer, effectively mapping salinity and infiltration risks. Furthermore, Khouni et al.^13^ utilized GIS-based IDW interpolation to produce detailed spatiotemporal pollution maps of Wadi El Bey, Tunisia, highlighting anthropogenic impacts and guiding local water quality interventions. Collectively, these studies have illustrated the growing relevance of GIS and IDW-based modeling approaches in hydro-environmental research and water quality monitoring.

Although spatial analysis is increasingly used in hydro-environmental research, GIS-based modeling has not yet been systematically applied to assess PTME patterns in Akdağ National Park, particularly through IDW interpolation. The ecological sensitivity of the park and the growing visitor pressure underline the value of such integrated monitoring efforts for establishing a robust baseline dataset and informing future conservation planning.

Akdağ National Park, spanning 15,933 hectares across Afyonkarahisar and Denizli provinces, hosts rich biological diversity and was officially designated a national park in 2024. Visitor numbers have risen markedly in recent years; however, the potential implications of this increase for local water bodies and wildlife have not been systematically examined. Accordingly, generating reliable empirical data on water quality and tracking its annual dynamics is essential for safeguarding the park’s ecological integrity.

This study represents the first year-round monitoring effort focusing on surface-water PTMEs within the park, the first statistical evaluation of their seasonal behavior, and the first GIS-based spatial characterization of water-quality variability. Given the ecological sensitivity and rising recreational use of the area, such an integrative approach offers critical baseline evidence to support long-term protection and adaptive management strategies.

This study aims to determine concentrations of PTMEs in surface water resources within the boundaries of Akdağ National Park and investigate their seasonal spatiotemporal variations. Within the scope of this study, the concentrations of aluminum (Al), arsenic (As), copper (Cu), boron (B), iron (Fe), calcium (Ca), chromium (Cr), lead (Pb), magnesium (Mg), manganese (Mn), nickel (Ni), and potassium (K) were determined in water samples collected from selected stations and evaluated in accordance with the criteria of the Türkiye Surface Water Quality Regulation. The results of this study provide an important data source for the development of sustainable strategies for monitoring and protecting water quality in the region.

## Materials and methods

### Study area and sampling stations

Based on the updated map of the region, field surveys were conducted, and the locations of the sampling stations were determined accordingly. During this process, consultations were held with researchers and staff from the Regional Directorate of National Parks to identify areas with continuous water availability throughout the year and select points representing the route of water flow within the national park.

**Fig. 1:**
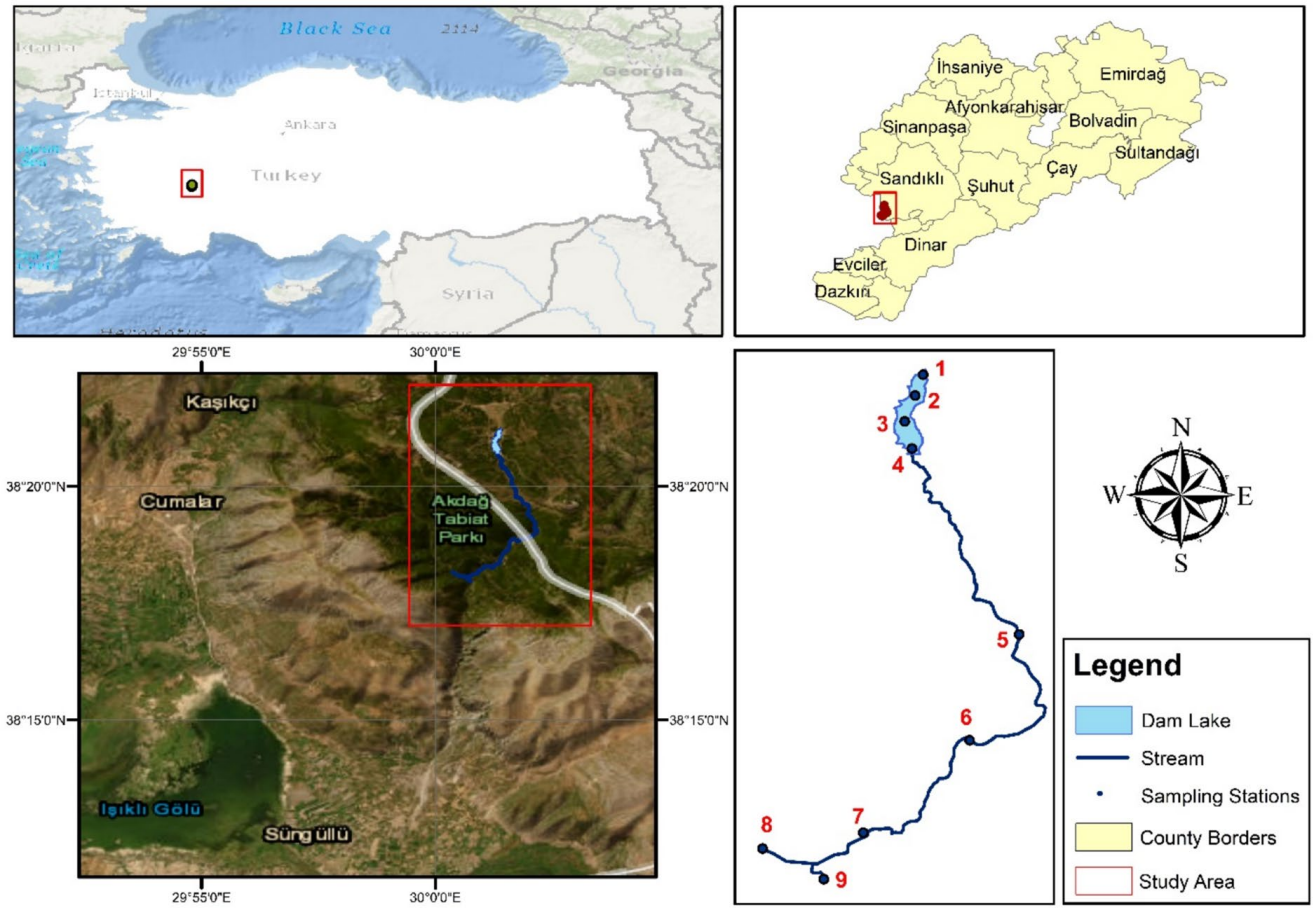
Study area and the spatial distribution of sampling stations. Map created in ArcMap 10.8. Basemap layer credits: Esri, GEBCO, NOAA, National Geographic, Garmin, HERE, Geonames.org, OpenStreetMap contributors, Maxar, Earthstar Geographics, and the GIS user community.

As shown in Figure 1, a total of nine sampling stations were established: one located on a perennial stream feeding the pond, three within the pond, and five along the perennial stream downstream of the pond. This way, the selected sampling stations were considered to best represent the overall characteristics of the park.

Water quality was monitored monthly over a 12-month period from October 2021 to September 2022 at the nine designated stations. Spatial and temporal variations of the measured parameters were evaluated to assess the water quality status of the area.

### Sampling and analysis

Water samples were collected from a depth of 15-20 cm below the surface in perennial streams, while samples from the pond were taken approximately 10-15 meters from the shore. The sampling procedure and sample volume followed the recommended guidelines of the Standard Methods for the Examination of Water and Wastewater^14^, ensuring consistency with internationally accepted protocols. The samples were collected rapidly by considering the distances between stations to minimize the possibility of different results caused by temperature fluctuations during sampling. While 12 sampling campaigns were planned between October 2021 and September 2022, water samples could not be collected from some stations in February and March 2022 due to adverse weather conditions and geographical constraints. For sample collection, 1000 mL polyethylene (PET) bottles pre-treated with a 1:1 HNO_3_-water solution and thoroughly rinsed with ultrapure water were used to prevent possible contamination at the beginning of the study. During sampling, the bottle cap was opened just before immersion, and the bottle was placed vertically into the water. To allow the sample to be collected from the desired depth, the mouth of the bottle was turned upward after filling, and the bottle was then capped. The collected samples were transported to the laboratory immediately.

### Elemental analysis (ICP-MS)

The elemental analyses were conducted on 11 November 2022 at the Atatürk University Eastern Anatolia High Technology Application and Research Center (DAYTAM) using an Agilent 7800 inductively coupled plasma-mass spectrometer (ICP-MS, Agilent Technologies, USA). The instrument consists of two units, inductively coupled plasma (ICP) and mass spectrometry (MS), which enable ionization and subsequent mass/charge (m/z) separation of elements. The concentrations of Al, As, Cu, B, Fe, Ca, Cr, Pb, Mg, Mn, Ni, and K were determined in the water samples.

The analyses were carried out by the accredited DAYTAM laboratory (ISO/IEC 17025 certified). Quality assurance and quality control (QA/QC) procedures included calibration with multi-element standards, routine analysis of certified reference material (NIST SRM 1643f), and the use of helium collision mode to minimize possible spectral interferences (e.g., Ar⁺, ArH⁺, CO₂⁺). The analytical precision and recovery rates reported by the laboratory were within ±5% and 92-107%, respectively.

### Statistical analysis

The data were analyzed using the Statistical Package for the Social Sciences (SPSS) 26.0 software. For the concentrations of Al, As, Cu, B, Fe, Ca, Cr, Pb, Mg, Mn, Ni, and K obtained from nine different stations during the fall, winter, spring, and summer seasons, mean ± standard deviation (Mean ± SD), median (Med), and minimum-maximum (Min-Max) values were calculated.

Since the sample size for each water quality parameter was relatively small (below 30), the use of non-parametric tests was deemed more appropriate for the analysis. Seasonal variations in water quality parameters at each station were analyzed using the Friedman Test. Differences in water quality parameters between stations within each season were evaluated using the Kruskal-Wallis H Test. In cases where significant differences were detected, the paired multiple comparison tests were applied to determine the sources of these differences. Throughout the study, the levels of statistical significance were taken as p<0.05 and p<0.01.

### Spatial interpolation

Firstly, the coordinate values of the obtained measurement points and the values of the examined PTMEs parameters were transferred to the GIS environment. It was used ArcGIS software. In the second stage, spatial distribution maps were created using the IDW method. The interpolation method is defined as the estimation of values belonging to unknown locations with the help of sample points. Environmental data can be interpolated using three principal approaches: classical statistical, deterministic, and geostatistical techniques. Deterministic interpolation techniques, on the other hand, aim to construct a continuous prediction surface from measured data based either on a similarity measure -such as inverse distance weighting- or on a smoothing function, as in radial basis functions. These methods vary depending on the characteristics of the study area and the expected surface form. Deterministic approaches are commonly grouped into exact and inexact methods according to whether estimated values replicate the observed data^15^^,^^16^.

The IDW method -a deterministic interpolation technique- is one of the most frequently used interpolation methods^17^. With the aid of sample points whose values are known, the IDW method determines the values of unsampled points. The magnitude and distance of the known points determine the predicted values, and the farther away the points are, the less significant and influential they are on the cell to be estimated^18^. The method is also widely used in studies on water parameters. For this reason, the IDW interpolation method was used in this study. While creating a map, the raw data are often arranged on a regular grid or occasionally dispersed haphazardly around a region, and interpolations are made on a denser regular grid. This approach uses a linear function of the distance between sets of point data and the points that need to be forecasted to calculate weights^19^.IDW utilizes the measured values around the prediction location to forecast a value for any unmeasured location. The predicted value from those farther away are influenced more by the measured values nearest to the prediction location. Therefore, IDW makes the assumption that the location-based influence of each measured point decreases with distance. The term “inverse distance weighted” comes from the fact that the method gives closer points a higher weight than those that are farther away^20^.The general equation is: $\hat{\mathrm{Z} }\left({x}_{0}\right)\frac{\sum_{i=1}^{n}z({x}_{i}){d}_{i0}^{-r}}{\sum_{i=1}^{n}{d}_{i0}^{-r}}$ (1) In equation 1, ${x}_{0}$: prediction location, ${x}_{i}$: observation location, ${d}_{i0}$: distance, and r: upper limit that determines the assigned range. As the upper limit increases, it is understood that the similarity between the predictions and the nearest observations is greater^21^^,^^22^.

During the interpolation process, several key parameters were defined to control the accuracy and structure of the resulting surface. The power (p) parameter determined the degree to which distance influenced the weighting function, with higher values giving greater importance to nearby points. The search radius specified the maximum distance within which sample points were considered for each estimation, thereby shaping the spatial extent of the interpolation. Additionally, the number of neighboring points used in each calculation was set to ensure stable and reliable predictions. Finally, the grid resolution, representing the cell size of the output raster, was selected to balance the level of detail with computational efficiency.

## Results

Elemental concentrations were monitored over a 12-month period at nine sampling stations in Akdağ Dam Lake and its tributaries. The dataset includes elements of environmental and toxicological importance -Al, As, Cu, B, Fe, Ca, Cr, Pb, Mg, Mn, Ni, and K -and their seasonal variations and spatial differences were evaluated based on four seasonal averages. Full seasonal descriptive statistics are available in Supplementary Material (Tables S1-S12).

**Fig. 2:**
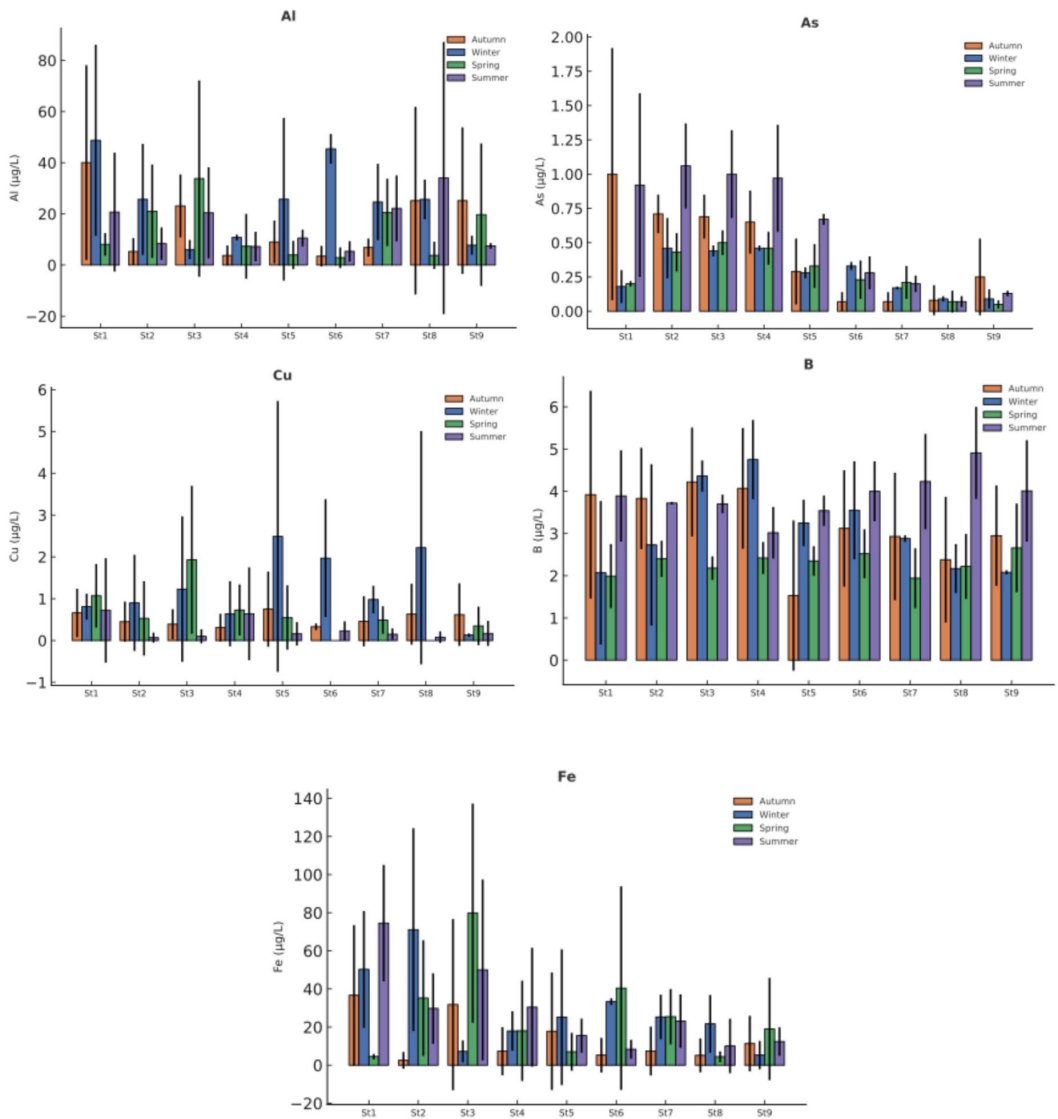
Seasonal variation of potentially toxic metal(loid)s (PTMEs) in water samples (µg/L, Mean ± SD) -Part I (Al, As, Cu, B, Fe).

As illustrated in Figure 2, the concentrations of Al, As, Cu, B, and Fe showed low-to-moderate variability across stations and seasons. Among these elements, Al, Cu, B, and Fe did not exhibit statistically significant seasonal variation (p > 0.05), indicating relatively stable geochemical behavior in both the dam lake and inflowing streams.As, however, displayed si2gnificant spatial variation during winter, spring, and summer (p < 0.05). Despite this spatial heterogeneity, As concentrations consistently remained at low levels across the monitored sites. The overall distribution patterns suggest that natural sources and background geological contributions dominate the occurrence of these metal(loid)s in the system.

**Fig. 3:**
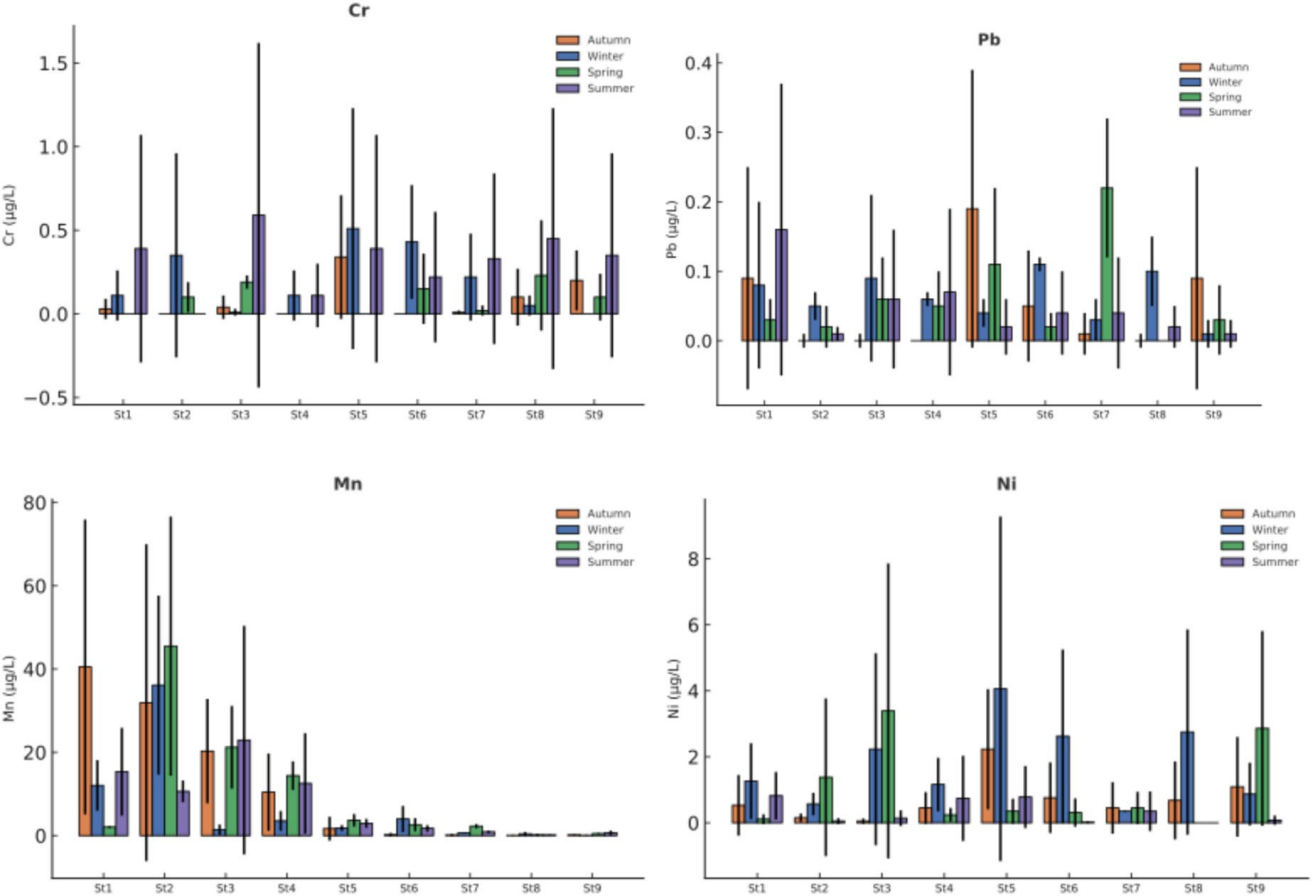
Seasonal variation of potentially toxic metal(loid)s (PTMEs) in water samples (µg/L, Mean ± SD) - Part II (Cr, Pb, Mn, Ni).

The seasonal profiles of Cr, Pb, Mn, and Ni are presented in Figure 3. Most of these elements showed no statistically significant seasonal differences (p > 0.05), indicating limited temporal fluctuations. Manganese (Mn) was the only element in this group that demonstrated significant spatial variation during winter, spring, and summer (p < 0.05). The elevated Mn values at certain stations appear to reflect localized geochemical or hydrological influences, potentially associated with sediment-water interactions or redox-sensitive mobilization processes.Overall, Cr, Pb, Mn, and Ni concentrations remained low and showed no indication of widespread contamination.

**Fig. 4:**
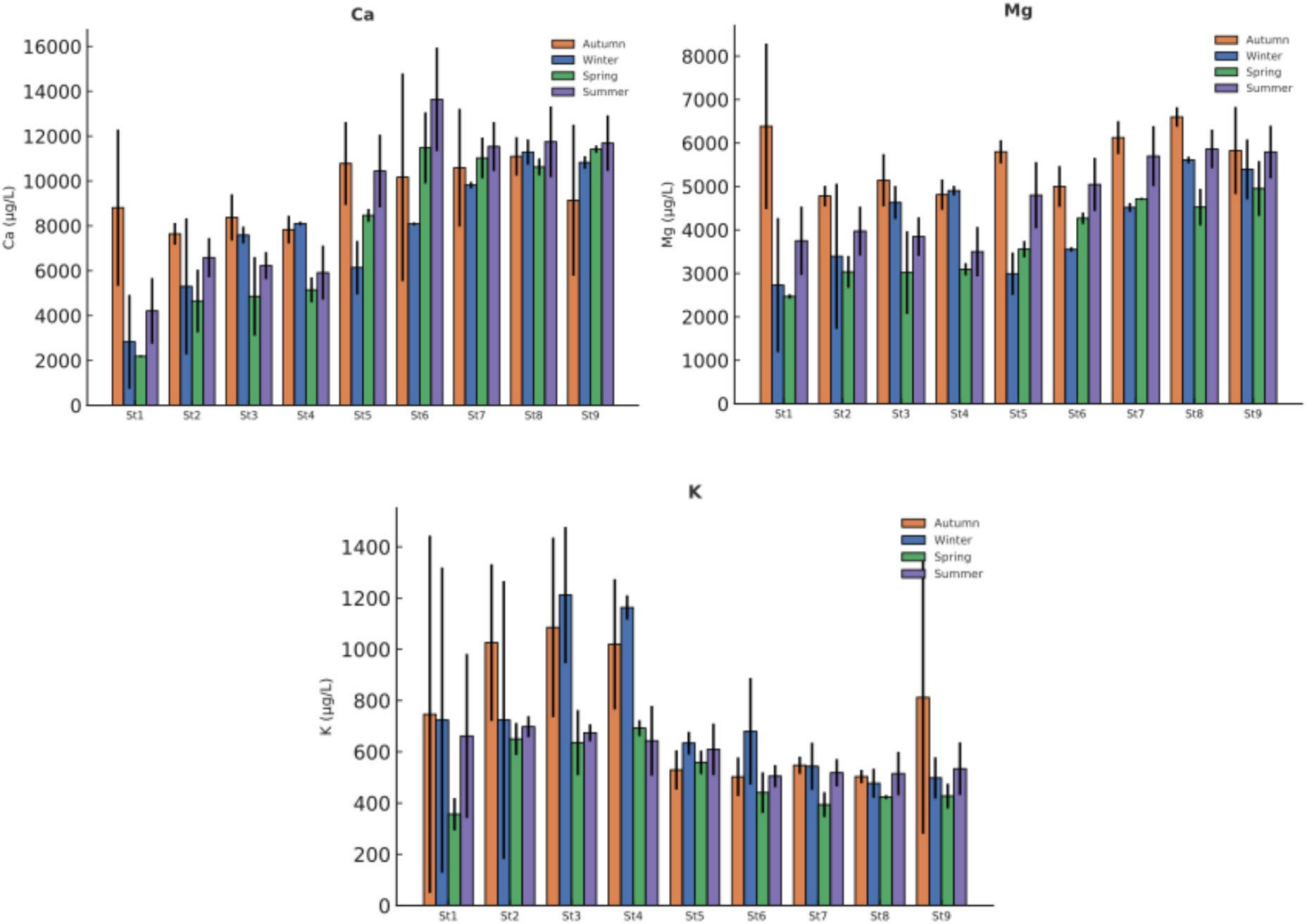
Seasonal variation of major cations in water samples (Ca, Mg, and K; mg/L, Mean ± SD).

Major cations displayed distinct patterns compared with PTMEs. As shown in Figure 4 Ca and Mg exhibited significant spatial variation during winter and spring (p < 0.05), likely driven by lithological and geochemical characteristics of the watershed. All three major elements -Ca, Mg, and K- occurred at markedly higher concentrations than trace metals, reflecting their natural abundance in the regional geology. K remained relatively consistent across seasons and stations, pointing to a more uniform geochemical distribution within the basin.

### Spatial analysis

The parts per billion (µg/L) values of all elemental concentration parameters measured for 12 months were averaged over four seasons, and the distribution maps of all parameters were created using the IDW method. The interpolation parameters were set to ensure accurate and reliable results. The power value was set to 2, which is commonly applied in similar studies to achieve a balanced weighting of distances. The number of neighbors was defined as 2, allowing both the preceding and succeeding data points to contribute to each estimation. A grid resolution of 4 mm was chosen to produce high-precision results. These parameter settings were carefully applied and documented to maintain methodological transparency and reproducibility.

**Fig. 5:**
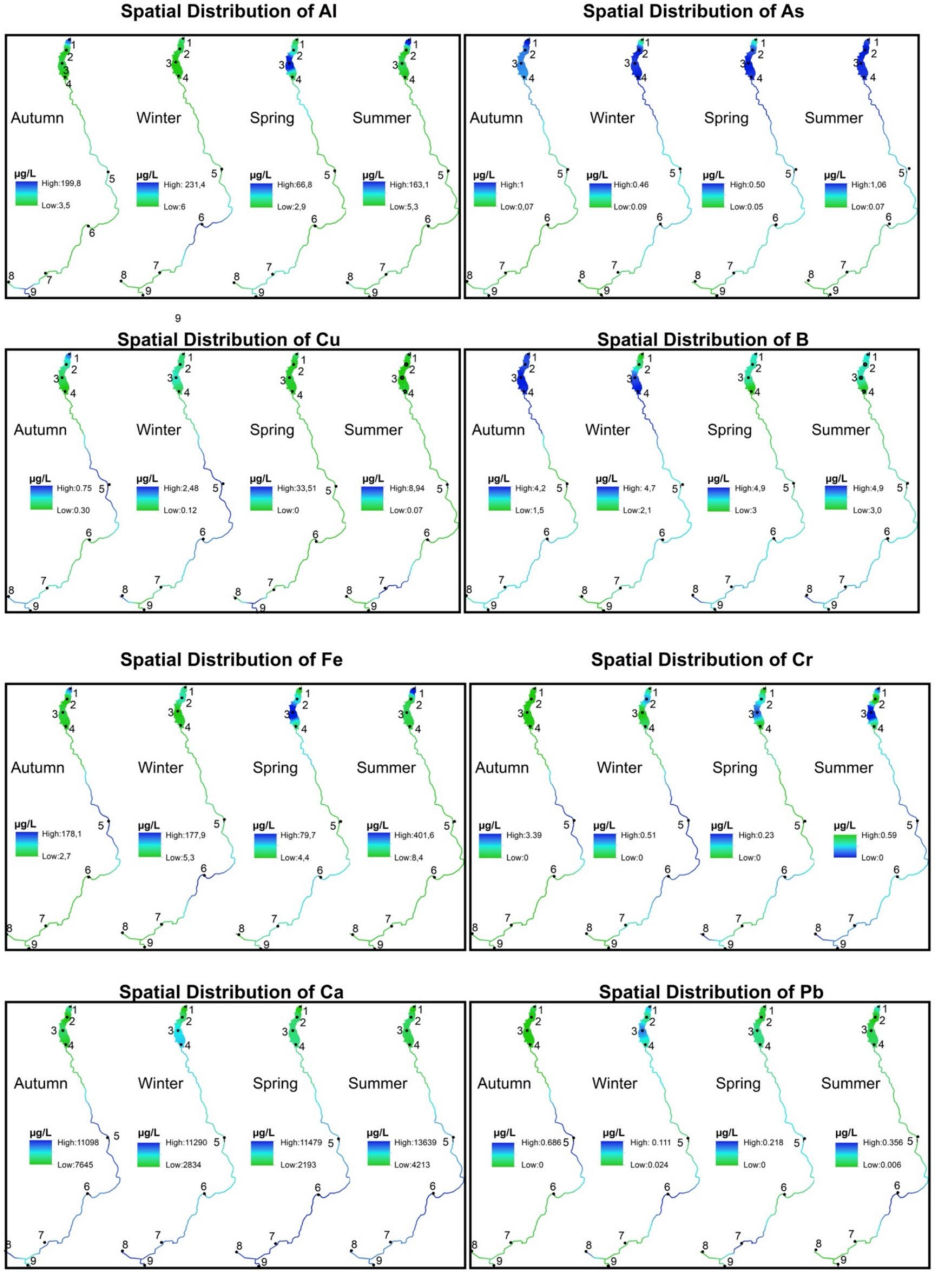

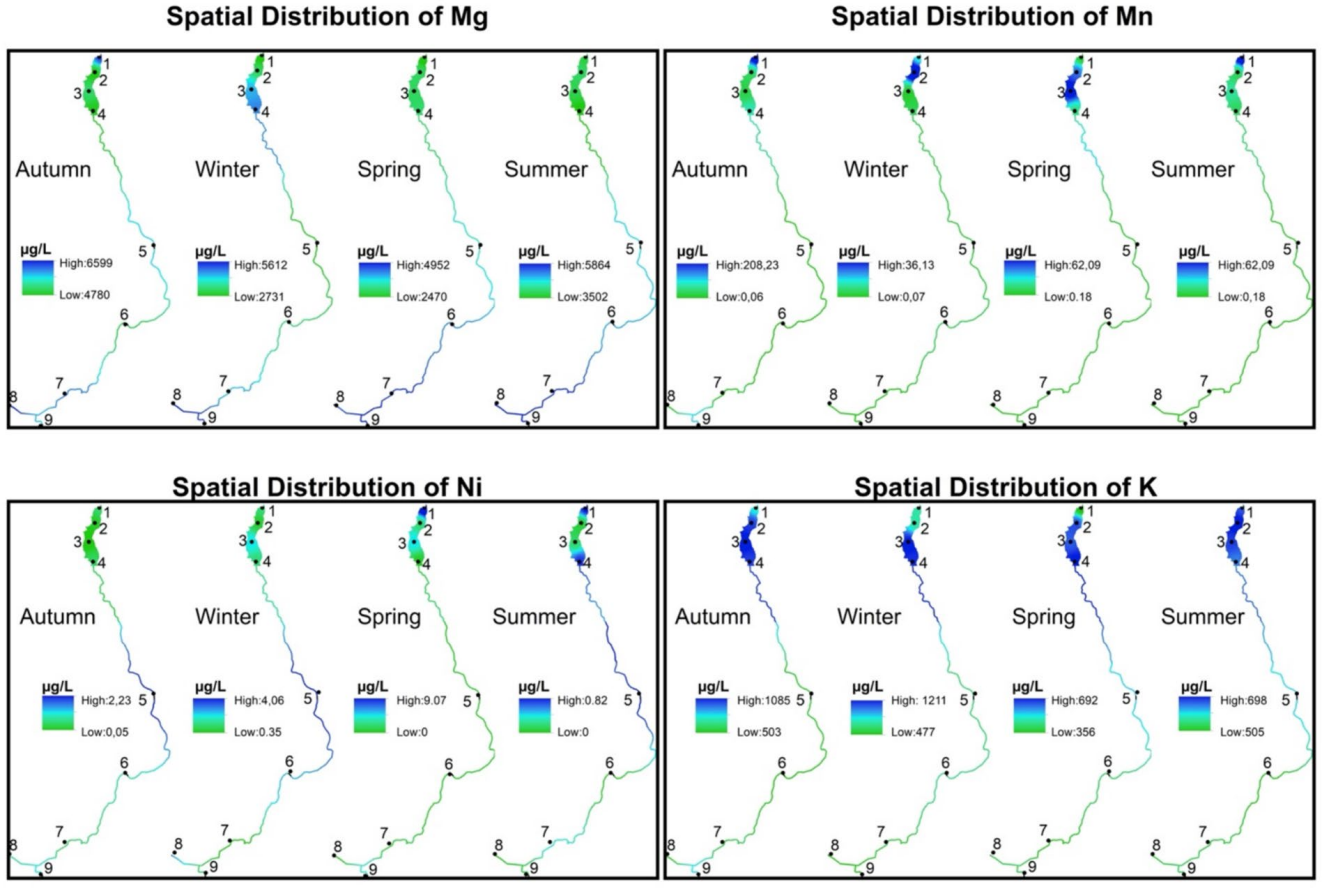
Seasonal spatial distribution maps of PTMEs generated using IDW interpolation. Map created in ArcMap 10.8. Basemap layer credits: Esri, GEBCO, NOAA, National Geographic, Garmin, HERE, Geonames.org, OpenStreetMap contributors, Maxar, Earthstar Geographics, and the GIS user community. Green areas indicate lower concentrations, while blue areas represent higher concentrations.

Stations 1, 2, 3 and 4 are shown spatially within the dam lake, and stations 5, 6, 7, 8 and 9 are shown linearly on the stream. The values of all distributions are shown by stretching on the color palette from green to blue. In this study, no classification was applied to the raster data. Instead, a stretch enhancement was used to improve visual interpretability by expanding the range of pixel values. Through this method, the raw pixel values were linearly rescaled within a defined minimum-maximum range, producing more distinguishable color variations within the green-blue gradient. This approach allowed the data to be represented with a continuous color ramp rather than discrete class boundaries. Accordingly, green areas represent places with low µg/L values, and blue areas represent places with high µg/L values. The highest and lowest values of each parameter are presented on the maps. The spatial distributions of all parameters are given for the four seasons in Figure 5.

The spatial distribution of potentially toxic and major elements (PTMEs) across the nine sampling stations showed clear seasonal and locational differences in Akdağ Dam Lake and its inflowing streams. Aluminum concentrations were generally low, with slight accumulations in upstream sections during fall and in the dam lake during winter and summer, remaining below regulatory limits. Arsenic accumulated consistently in the dam lake and nearby streams, with higher levels in winter and spring, but still below the WHO and EU threshold of 10 µg/L. Copper was more concentrated in stream sections than in the lake, peaking in midstream zones during fall and winter and shifting upstream in spring and summer. Boron accumulated mainly in the dam lake and adjacent streams, particularly in winter and summer, while lower levels occurred in the inner lake during spring.

Iron, manganese, chromium, and nickel showed more distinct spatial and temporal variations. Iron concentrations were elevated in the downstream lake and central streams during fall and winter but shifted toward mid-lake and downstream zones in spring and summer. Manganese tended to accumulate in the dam lake throughout all seasons, with higher levels in the mid-lake in spring. Chromium was concentrated in midstream zones across seasons, while nickel peaked in midstream areas except during spring, when it increased in downstream sections.

Calcium, magnesium, and potassium followed patterns typical of major elements. Calcium concentrations were higher in upstream sections and lower in the dam lake, whereas magnesium was enriched upstream and moderately elevated downstream during fall and winter. Potassium showed a relatively uniform distribution, with slightly higher levels in the dam lake during fall and winter. Lead concentrations remained low overall, except for a slight increase in the dam lake during winter and midstream sections during fall. All Pb values were below regulatory limits, indicating minimal anthropogenic influence.

## Discussion

This study provides the first systematic assessment of seasonal and spatial variations in potentially toxic and major elements (PTMEs) in the surface waters of Akdağ National Park. Overall, elemental concentrations- Al, As, Cu, B, Fe, Cr, Pb, Mn, Ni, Ca, Mg, and K -remained within TS 266 and EU WFD limits, generally corresponding to the A1-A2 water-quality classes. The limited seasonal variation suggests that hydrological processes typical of semi-arid regions, including precipitation, snowmelt, and evaporation, govern short-term fluctuations in water chemistry, consistent with previous findings from other freshwater systems in Türkiye^1^^,^^3^.

Spatial heterogeneity was more evident for Mn and Fe, whose elevated values at specific stations likely reflect redox-driven sediment-water interactions and natural geochemical mobilization. Similar geogenic controls have been reported in Eastern European and Balkan catchments, where Fe and Mn commonly dominate surface-water chemistry under low anthropogenic pressure^23,24^. These patterns are also consistent with regional assessments from Greece, where natural lithology and sediment-water dynamics strongly influence trace-metal behavior^25^.

The deterministic IDW interpolation used in this study, aligned with established GIS-based approaches^19^^,^^13^, further helped distinguish natural spatial patterns from potential localized influences. Regional and European assessments show that many surface waters exhibit elevated Pb, Ni, and Cr concentrations under strong industrial and agricultural pressure. In contrast, Pb concentrations in Akdağ remained below 1 µg/L across all seasons, and the modest increases in Mn and Fe are more plausibly linked to sediment-derived natural processes than to anthropogenic inputs.

Element-specific comparisons also support this conclusion. Al, Cu, B remained consistently low, reflecting background geochemical conditions. As showed mild spatial variation yet remained within EU guideline levels. Cr and Ni exhibited stable, low concentrations compared with those typically observed in mining-impacted water bodies of the Balkans and Eastern Europe^23,24^. Ca and Mg increased during winter and spring, supporting the presence of a carbonate-bicarbonate buffering system characteristic of naturally regulated hydrogeochemical settings.

Although geological mapping was not available for this study, the combined evidence -low contaminant levels, stable seasonal patterns, and spatial variability consistent with natural processes- indicates that water quality in Akdağ National Park is primarily shaped by geogenic controls. Compliance with international benchmarks and clear contrasts with polluted reference systems across the Balkans and southern Europe^23–25^ suggest that the reservoir currently maintains a low-risk chemical profile. Continued monitoring will remain important for detecting emerging pressures and supporting the long-term protection of this ecologically sensitive region.

## Conclusion

This year-long assessment shows that the concentrations of potentially toxic and major elements (PTMEs) in the surface waters of Akdağ National Park remain within national (TS 266) and international (WHO, EU) limits. Most elements were consistently low, while seasonal differences observed for As, Mn, Ca, and Mg likely reflect natural geological and hydrological variability rather than external pollution. Importantly, none of the parameters exceeded guideline thresholds, indicating no immediate risk to ecological or human health. Although overall water quality appears stable, stations with slightly elevated values during winter and spring should continue to be monitored to detect potential changes early. Maintaining regular, targeted monitoring will be essential to safeguard the park’s favorable water-quality status as environmental conditions evolve.

## Supplementary Information

Supplementary Information.

## Declarations

### Responsibilities of authors

All authors have read, understood, and complied as applicable with the statement on “Ethical responsibilities of Authors” as found in the ınstructions for authors.
