# Supplementary material for: ICP-MS based seasonal and spatiotemporal evaluation of potentially toxic and major elements in surface waters of Akdağ National Park, Türkiye
Source: Sci Rep. 2026 Feb 13;16:8508. doi: 10.1038/s41598-026-35053-z (PMC12972310; doi:10.1038/s41598-026-35053-z)
Supplement: Supplementary file 1 — Supplementary Information. [file 41598_2026_35053_MOESM1_ESM.docx]

These supplementary tables contain full seasonal descriptive statistics (mean ± SD, median, min–max, and p-values) for all sampling stations.

Table 1. Seasonal Variation of Al Concentrations (µg/L, Mean ± SD)

| Station | Fall Mean ± SD (Med; Min–Max) | Winter Mean ± SD (Med; Min–Max) | Spring Mean ± SD (Med; Min–Max) | Summer Mean ± SD (Med; Min–Max) | p₁ |
| --- | --- | --- | --- | --- | --- |
| 1 | 40.05±38.06 (44.4; 0–75.74) | 48.76±37.35 (28.29; 26.12–91.88) | 8.07±4.42 (10.12; 2.99–11.09) | 20.7±23.24 (9.74; 4.96–47.4) | 0.457 |
| 2 | 5.28±5.23 (5.37; 0–10.47) | 25.67±21.70 (15.43; 10.98–50.59) | 21.03±18.28 (29.98; 0–33.12) | 8.36±6.36 (9.0; 1.71–14.38) | 0.241 |
| 3 | 23.11±12.3 (24.01; 10.39–34.94) | 6.07±3.75 (6.07; 3.42–8.73) | 33.79±38.37 (12.26; 11.02–78.09) | 20.39±17.84 (21.28; 2.12–37.76) | 0.241 |
| 4 | 3.74±3.92 (3.41; 0–7.83) | 10.77±1.13 (10.77; 9.97–11.57) | 7.32±12.67 (0.00; 0–21.95) | 7.22±5.79 (7.31; 1.38–12.97) | 0.615 |
| 5 | 9.02±8.4 (6.05; 2.51–18.5) | 25.70±31.81 (25.7; 3.2–48.19) | 3.92±5.54 (3.92; 0–7.83) | 10.55±3.26 (10.45; 7.35–13.87) | 0.241 |
| 6 | 3.45±4.03 (2.46; 0–7.88) | 45.41±5.82 (45.41; 41.3–49.53) | 2.86±4.04 (2.86; 0–5.72) | 5.32±4 (5.56; 1.21–9.2) | 0.145 |
| 7 | 6.86±3.51 (8.46; 2.83–9.28) | 24.64±14.93 (24.64; 14.08–35.2) | 20.56±13.19 (20.56; 11.23–29.88) | 22.15±12.9 (15.15; 14.25–37.03) | 0.241 |
| 8 | 25.17±36.67 (6.87; 1.25–67.39) | 25.65±7.75 (25.65; 20.17–31.13) | 3.78±5.34 (3.78; 0–7.56) | 34.03±53.22 (3.37; 3.23–95.48) | 0.753 |
| 9 | 25.17±28.64 (9.89; 7.4–58.2) | 7.74±3.70 (7.74; 5.12–10.35) | 19.69±27.85 (19.69; 0–39.39) | 7.47±1.14 (8.1; 6.16–8.16) | 0.615 |
| p₂ | 0.423 | 0.201 | 0.451 | 0.542 |  |

**p1**: Intra-station seasonal variation (Friedman Test) **p2**: Inter-station seasonal differences (Kruskal-Wallis H Test) *p<0.05; **p<0.01

Table 2. Seasonal Variation of As Concentrations (µg/L, Mean ± SD)

| Station | Fall Mean ± SD | Fall Median (Min.–Max.) | Winter Mean ± SD | Winter Median (Min.–Max.) | Spring Mean ± SD | Spring Median (Min.–Max.) | Summer Mean ± SD | Summer Median (Min.–Max.) | p1 |
| --- | --- | --- | --- | --- | --- | --- | --- | --- | --- |
| 1 | 1.00±0.92 | 1.20 (0–1.80) | 0.18±0.12 | 0.23 (0.04–0.27) | 0.20±0.02 | 0.21 (0.18–0.22) | 0.92±0.67 | 0.75 (0.35–1.66) | 0.241 |
| 2 | 0.71±0.14 | 0.68 (0.59–0.85) | 0.46±0.22 | 0.37 (0.31–0.72) | 0.43±0.14 | 0.36 (0.35–0.60) | 1.06±0.31 | 1.08 (0.75–1.36) | 0.06 |
| 3 | 0.69±0.16 | 0.68 (0.53–0.85) | 0.44±0.04 | 0.44 (0.41–0.47) | 0.50±0.09 | 0.47 (0.44–0.60) | 1.00±0.32 | 0.81 (0.81–1.37) | 0.187 |
| 4 | 0.65±0.23 | 0.67 (0.41–0.86) | 0.46±0.02 | 0.46 (0.44–0.48) | 0.46±0.12 | 0.46 (0.35–0.58) | 0.97±0.39 | 0.97 (0.59–1.36) | 0.112 |
| 5 | 0.29±0.24 | 0.17 (0.12–0.56) | 0.28±0.04 | 0.28 (0.25–0.31) | 0.33±0.16 | 0.33 (0.21–0.44) | 0.67±0.04 | 0.69 (0.63–0.69) | 0.284 |
| 6 | 0.07±0.07 | 0.06 (0–0.14) | 0.33±0.03 | 0.33 (0.31–0.35) | 0.23±0.14 | 0.23 (0.12–0.33) | 0.28±0.12 | 0.32 (0.15–0.38) | 0.392 |
| 7 | 0.07±0.07 | 0.07 (0–0.14) | 0.17±0.01 | 0.17 (0.17–0.17) | 0.21±0.12 | 0.21 (0.12–0.29) | 0.20±0.06 | 0.17 (0.15–0.26) | 0.494 |
| 8 | 0.08±0.11 | 0.04 (0–0.21) | 0.09±0.02 | 0.09 (0.08–0.11) | 0.07±0.08 | 0.07 (0.01–0.13) | 0.07±0.04 | 0.08 (0.03–0.11) | 0.896 |
| 9 | 0.25±0.28 | 0.12 (0.06–0.57) | 0.09±0.07 | 0.09 (0.04–0.14) | 0.05±0.03 | 0.05 (0.03–0.07) | 0.13±0.02 | 0.14 (0.11–0.14) | 0.615 |
| p2 | 0.070 |  | 0.041* | 2, 3, 4 > 8, 9 | 0.027* | 2, 3, 4, 5 > 8, 9 | 0.005** | 1, 2, 3, 4, 5 > 7, 8, 9 | |

**p1**: Intra-station seasonal variation (Friedman Test) **p2**: Inter-station seasonal differences (Kruskal-Wallis H Test) *p<0.05; **p<0.01

Table 3. Seasonal Variation of Cu Concentrations (µg/L, Mean ± SD)

| Station | Fall Mean ± SD | Fall Median (Min.–Max.) | Winter Mean ± SD | Winter Median (Min.–Max.) | Spring Mean ± SD | Spring Median (Min.–Max.) | Summer Mean ± SD | Summer Median (Min.–Max.) | p1 |
| --- | --- | --- | --- | --- | --- | --- | --- | --- | --- |
| 1 | 0.66±0.58 | 0.92 (0–1.06) | 0.81±0.31 | 0.63 (0.62–1.17) | 1.07±0.76 | 1.37 (0.20–1.64) | 0.72±1.25 | 0 (0–2.16) | 0.818 |
| 2 | 0.45±0.48 | 0.40 (0–0.96) | 0.90±1.15 | 0.28 (0.19–2.23) | 0.53±0.89 | 0.04 (0–1.56) | 0.07±0.12 | 0 (0–0.21) | 0.27 |
| 3 | 0.39±0.36 | 0.44 (0–0.72) | 1.23±1.74 | 1.23 (0–2.46) | 1.93±1.77 | 1.52 (0.40–3.88) | 0.10±0.17 | 0 (0–0.30) | 0.304 |
| 4 | 0.31±0.33 | 0.26 (0–0.66) | 0.64±0.78 | 0.64 (0.09–1.19) | 0.73±0.61 | 0.38 (0.38–1.43) | 0.64±1.11 | 0 (0–1.92) | 0.896 |
| 5 | 0.75±0.90 | 0.25 (0.22–1.79) | 2.49±3.24 | 2.49 (0.20–4.78) | 0.55±0.77 | 0.55 (0.01–1.10) | 0.16±0.28 | 0 (0–0.48) | 0.072 |
| 6 | 0.33±0.08 | 0.30 (0.28–0.42) | 1.97±1.41 | 1.97 (0.98–2.97) | 0±0 | 0 (0–0) | 0.23±0.23 | 0.24 (0–0.46) | 0.145 |
| 7 | 0.46±0.60 | 0.24 (0–1.14) | 0.98±0.33 | 0.98 (0.74–1.21) | 0.49±0.33 | 0.49 (0.25–0.72) | 0.15±0.14 | 0.19 (0–0.26) | 0.494 |
| 8 | 0.63±0.73 | 0.46 (0–1.43) | 2.22±2.79 | 2.22 (0.25–4.19) | 0±0 | 0 (0–0) | 0.08±0.14 | 0 (0–0.24) | 0.18 |
| 9 | 0.62±0.75 | 0.40 (0–1.45) | 0.13±0.04 | 0.13 (0.09–0.16) | 0.35±0.46 | 0.35 (0.02–0.67) | 0.17±0.30 | 0 (0–0.52) | 0.304 |
| p2 | 0.990 |  | 0.695 |  | 0.162 |  | 0.994 |  |  |

**p1**: Intra-station seasonal variation (Friedman Test) **p2**: Inter-station seasonal differences (Kruskal-Wallis H Test) *p<0.05; **p<0.01

Table 4. Seasonal Variation of B Concentrations (µg/L, Mean ± SD)

| Station | Fall Mean ± SD | Fall Median (Min.–Max.) | Winter Mean ± SD | Winter Median (Min.–Max.) | Spring Mean ± SD | Spring Median (Min.–Max.) | Summer Mean ± SD | Summer Median (Min.–Max.) | p1 |
| --- | --- | --- | --- | --- | --- | --- | --- | --- | --- |
| 1 | 3.92±2.46 | 4.53 (1.22–6.02) | 2.07±1.70 | 2.49 (0.19–3.52) | 1.99±0.76 | 1.75 (1.37–2.84) | 3.89±1.08 | 3.86 (2.83–4.99) | 0.334 |
| 2 | 3.83±1.20 | 4.37 (2.46–4.67) | 2.73±1.91 | 3.48 (0.55–4.14) | 2.40±0.43 | 2.26 (2.06–2.89) | 3.72±0.03 | 3.72 (3.69–3.76) | 0.122 |
| 3 | 4.22±1.29 | 4.48 (2.82–5.35) | 4.36±0.37 | 4.36 (4.10–4.62) | 2.18±0.28 | 2.10 (1.96–2.50) | 3.70±0.22 | 3.83 (3.45–3.84) | 0.145 |
| 4 | 4.07±1.43 | 4.73 (2.43–5.05) | 4.75±0.94 | 4.75 (4.08–5.41) | 2.42±0.38 | 2.27 (2.13–2.85) | 3.02±0.61 | 3.17 (2.34–3.54) | 0.145 |
| 5 | 1.53±1.78 | 1.11 (0–3.49) | 3.25±0.55 | 3.25 (2.87–3.64) | 2.35±0.35 | 2.35 (2.10–2.59) | 3.54±0.36 | 3.69 (3.12–3.80) | 0.145 |
| 6 | 3.12±1.38 | 2.44 (2.21–4.70) | 3.55±1.16 | 3.55 (2.73–4.37) | 2.52±0.58 | 2.52 (2.11–2.93) | 4.00±0.71 | 3.72 (3.48–4.80) | 0.615 |
| 7 | 2.93±1.51 | 2.44 (1.74–4.63) | 2.88±0.08 | 2.88 (2.83–2.94) | 1.94±0.71 | 1.94 (1.44–2.44) | 4.23±1.13 | 3.88 (3.31–5.49) | 0.494 |
| 8 | 2.38±1.49 | 2.44 (0.86–3.83) | 2.17±0.58 | 2.17 (1.76–2.57) | 2.22±0.77 | 2.22 (1.67–2.76) | 4.91±1.09 | 5.40 (3.66–5.66) | 0.308 |
| 9 | 2.95±1.19 | 2.76 (1.86–4.22) | 2.08±0.05 | 2.08 (2.05–2.12) | 2.66±1.05 | 2.66 (1.92–3.40) | 4.01±1.20 | 3.37 (3.26–5.40) | 0.615 |
| p2 | 0.497 |  | 0.133 |  | 0.883 |  | 0.489 |  |  |

**p1**: Intra-station seasonal variation (Friedman Test) **p2**: Inter-station seasonal differences (Kruskal-Wallis H Test) *p<0.05; **p<0.01

Table 5. Seasonal Variation of Fe Concentrations (µg/L, Mean ± SD)

| Station | Fall Mean ± SD | Fall Median (Min.–Max.) | Winter Mean ± SD | Winter Median (Min.–Max.) | Spring Mean ± SD | Spring Median (Min.–Max.) | Summer Mean ± SD | Summer Median (Min.–Max.) | p1 |
| --- | --- | --- | --- | --- | --- | --- | --- | --- | --- |
| 1 | 36.65±36.83 | 36.30 (0–73.65) | 50.23±30.68 | 64.33 (15.03–71.32) | 4.48±1.45 | 3.77 (3.53–6.15) | 74.52±30.53 | 63.64 (50.92–109) | 0.122 |
| 2 | 2.53±4.39 | 0 (0–7.60) | 71.09±53.23 | 66.10 (20.54–126.64) | 35.17±30.37 | 34.04 (5.38–66.09) | 29.71±18.52 | 28.68 (11.74–48.73) | 0.086 |
| 3 | 31.76±44.91 | 31.76 (0–63.52) | 7.20±5.78 | 7.20 (3.11–11.29) | 79.74±57.47 | 85.26 (19.71–134.26) | 50.00±47.46 | 43.51 (6.13–100.37) | 0.241 |
| 4 | 7.30±12.64 | 0 (0–21.90) | 17.93±10.35 | 17.93 (10.61–25.25) | 18.00±26.33 | 5.77 (0–48.22) | 30.39±31.20 | 20.84 (5.07–65.25) | 0.776 |
| 5 | 17.80±30.83 | 0 (0–53.40) | 25.17±35.60 | 25.17 (0–50.35) | 7.03±9.94 | 7.03 (0–14.06) | 15.52±8.95 | 17.94 (5.61–23.02) | 0.764 |
| 6 | 5.24±9.08 | 0 (0–15.72) | 33.36±1.78 | 33.36 (32.10–34.61) | 40.40±53.41 | 40.40 (2.64–78.17) | 8.35±4.97 | 9.15 (3.03–12.87) | 0.615 |
| 7 | 7.42±12.84 | 0 (0–22.25) | 25.30±11.69 | 25.30 (17.03–33.57) | 25.45±14.52 | 25.45 (15.19–35.72) | 23.14±14.03 | 21.39 (10.08–37.96) | 0.615 |
| 8 | 5.13±8.89 | 0 (0–15.40) | 21.66±15.16 | 21.66 (10.94–32.38) | 4.39±2.87 | 4.39 (2.36–6.42) | 10.08±14.30 | 3.80 (0–26.45) | 0.852 |
| 9 | 11.33±14.57 | 6.22 (0–27.76) | 5.28±7.46 | 5.28 (0–10.55) | 18.97±26.82 | 18.97 (0–37.94) | 12.40±7.49 | 15.11 (3.94–18.16) | 0.776 |
| p2 | 0.887 |  | 0.237 |  | 0.421 |  | 0.147 |  |  |

**p1**: Intra-station seasonal variation (Friedman Test) **p2**: Inter-station seasonal differences (Kruskal-Wallis H Test) *p<0.05; **p<0.01

Table 6. Seasonal Variation of Ca Concentrations (µg/L, Mean ± SD)

| Station | Fall Mean ± SD | Fall Median (Min.–Max.) | Winter Mean ± SD | Winter Median (Min.–Max.) | Spring Mean ± SD | Spring Median (Min.–Max.) | Summer Mean ± SD | Summer Median (Min.–Max.) | p1 |
| --- | --- | --- | --- | --- | --- | --- | --- | --- | --- |
| 1 | 8811.66±3482.80 | 7923.88 (5858.67–12652.42) | 2833.71±2092.95 | 2805.35 (755.09–4940.69) | 2192.10±34.26 | 2196.93 (2155.68–2223.69) | 4212.73±1463.37 | 4178.33 (2766.86–5693.00) | 0.072 |
| 2 | 7645.10±488.11 | 7887.19 (7083.27–7964.84) | 5301.35±3033.97 | 5697.19 (2088.90–8117.97) | 4653.01±1397.57 | 5402.42 (3040.57–5516.04) | 6588.08±873.12 | 6801.30 (5628.11–7334.84) | 0.172 |
| 3 | 8376.95±1031.84 | 8748.83 (7210.72–9171.30) | 7591.50±377.54 | 7591.50 (7324.54–7858.46) | 4862.23±1752.24 | 5539.19 (2872.50–6175.01) | 6229.32±607.23 | 6067.54 (5719.37–6901.06) | 0.145 |
| 4 | 7833.81±618.50 | 7928.54 (7173.41–8399.48) | 8106.39±78.99 | 8106.39 (8050.54–8162.25) | 5147.23±559.81 | 5436.86 (4501.94–5502.88) | 5917.35±1206.32 | 5751.22 (4802.71–7198.13) | 0.112 |
| 5 | 10778.98±1850.10 | 11636.38 (8655.73–12044.84) | 6138.66±1194.40 | 6138.66 (5294.09–6983.22) | 8470.98±287.62 | 8470.98 (8267.60–8674.36) | 10444.93±1620.29 | 11312.66 (8575.57–11446.55) | 0.145 |
| 6 | 10167.08±4629.92 | 8196.30 (6848.62–15456.33) | 8091.16±63.95 | 8091.16 (8045.94–8136.37) | 11479.31±1586.65 | 11479.31 (10357.38–12601.24) | 13639.13±2306.20 | 14806.18 (10982.67–15128.55) | 0.308 |
| 7 | 10594.83±2624.69 | 10630.61 (7952.44–13201.45) | 9818.87±148.03 | 9818.87 (9714.20–9923.54) | 11027.58±911.21 | 11027.58 (10383.26–11671.91) | 11534.35±1094.12 | 11176.10 (10664.27–12762.69) | 0.241 |
| 8 | 11098.08±857.47 | 10987.99 (10300.97–12005.28) | 11290.17±563.31 | 11290.17 (10891.85–11688.49) | 10628.78±379.74 | 10628.78 (10360.27–10897.30) | 11748.37±1577.16 | 11997.42 (10061.50–13186.19) | 1 |
| 9 | 9143.17±3365.41 | 8541.57 (6119.13–12768.81) | 10827.30±283.39 | 10827.30 (10626.91–11027.68) | 11418.58±162.83 | 11418.58 (11303.44–11533.72) | 11687.33±1236.73 | 12297.38 (10264.11–12500.51) | 0.896 |
| p2 | 0.441 |  | 0.031* | 8,9 > 2,3,4,5,6,7 > 1 | 0.016* | 6,7,8,9 > 1 | 0.007** | 5,6,7,8,9 > 1,4 | |

**p1**: Intra-station seasonal variation (Friedman Test) **p2**: Inter-station seasonal differences (Kruskal-Wallis H Test) *p<0.05; **p<0.01

Table 7. Seasonal Variation of Cr Concentrations (µg/L, Mean ± SD)

| Station | Fall Mean ± SD | Fall Median (Min.–Max.) | Winter Mean ± SD | Winter Median (Min.–Max.) | Spring Mean ± SD | Spring Median (Min.–Max.) | Summer Mean ± SD | Summer Median (Min.–Max.) | p1 |
| --- | --- | --- | --- | --- | --- | --- | --- | --- | --- |
| 1 | 0.03±0.06 | 0 (0–0.10) | 0.11±0.15 | 0.06 (0–0.28) | 0±0 | 0 (0–0) | 0.39±0.68 | 0 (0–1.17) | 0.608 |
| 2 | 0±0 | 0 (0–0) | 0.35±0.61 | 0 (0–1.06) | 0.10±0.09 | 0.13 (0–0.17) | 0±0 | 0 (0–0) | 0.274 |
| 3 | 0.04±0.07 | 0 (0–0.12) | 0.01±0.02 | 0.01 (0–0.02) | 0.19±0.04 | 0.20 (0.15–0.22) | 0.59±1.03 | 0 (0–1.78) | 0.29 |
| 4 | 0±0 | 0 (0–0) | 0.11±0.15 | 0.11 (0–0.21) | 0±0 | 0 (0–0) | 0.11±0.19 | 0 (0–0.32) | 0.392 |
| 5 | 0.34±0.37 | 0.28 (0–0.73) | 0.51±0.72 | 0.51 (0–1.02) | 0±0 | 0 (0–0) | 0.39±0.68 | 0 (0–1.18) | 0.615 |
| 6 | 0±0 | 0 (0–0) | 0.43±0.34 | 0.43 (0.19–0.67) | 0.15±0.21 | 0.15 (0–0.30) | 0.22±0.39 | 0 (0–0.67) | 0.304 |
| 7 | 0.01±0.01 | 0 (0–0.02) | 0.22±0.26 | 0.22 (0.03–0.41) | 0.02±0.03 | 0.02 (0–0.04) | 0.33±0.51 | 0.07 (0–0.92) | 0.776 |
| 8 | 0.10±0.17 | 0 (0–0.29) | 0.05±0.06 | 0.05 (0–0.09) | 0.23±0.33 | 0.23 (0–0.46) | 0.45±0.78 | 0 (0–1.34) | 0.861 |
| 9 | 0.20±0.18 | 0.27 (0–0.34) | 0±0 | 0 (0–0) | 0.10±0.14 | 0.10 (0–0.19) | 0.35±0.61 | 0 (0–1.06) | 0.418 |
| p2 | 0.351 |  | 0.705 |  | 0.248 |  | 0.973 |  |  |

**p1**: Intra-station seasonal variation (Friedman Test) **p2**: Inter-station seasonal differences (Kruskal-Wallis H Test) *p<0.05; **p<0.01

Table 8. Seasonal Variation of Pb Concentrations (µg/L, Mean ± SD)

| Station | Fall Mean ± SD | Fall Median (Min.–Max.) | Winter Mean ± SD | Winter Median (Min.–Max.) | Spring Mean ± SD | Spring Median (Min.–Max.) | Summer Mean ± SD | Summer Median (Min.–Max.) | p1 |
| --- | --- | --- | --- | --- | --- | --- | --- | --- | --- |
| 1 | 0.09±0.16 | 0 (0–0.27) | 0.08±0.12 | 0.02 (0.01–0.21) | 0.03±0.03 | 0.01 (0–0.06) | 0.16±0.21 | 0.07 (0–0.40) | 0.67 |
| 2 | 0±0.01 | 0 (0–0.01) | 0.05±0.02 | 0.05 (0.03–0.06) | 0.02±0.03 | 0 (0–0.05) | 0.01±0.01 | 0 (0–0.02) | 0.086 |
| 3 | 0±0.01 | 0 (0–0.01) | 0.09±0.12 | 0.09 (0–0.18) | 0.06±0.06 | 0.03 (0.02–0.12) | 0.06±0.10 | 0 (0–0.17) | 0.753 |
| 4 | 0±0 | 0 (0–0) | 0.06±0.01 | 0.06 (0.05–0.06) | 0.05±0.05 | 0.04 (0–0.11) | 0.07±0.12 | 0 (0–0.20) | 0.443 |
| 5 | 0.19±0.20 | 0.17 (0–0.40) | 0.04±0.02 | 0.04 (0.02–0.06) | 0.11±0.11 | 0.11 (0.03–0.19) | 0.02±0.04 | 0 (0–0.07) | 0.308 |
| 6 | 0.05±0.08 | 0 (0–0.14) | 0.11±0.01 | 0.11 (0.11–0.12) | 0.02±0.02 | 0.02 (0.01–0.04) | 0.04±0.06 | 0 (0–0.11) | 0.12 |
| 7 | 0.01±0.03 | 0 (0–0.04) | 0.03±0.03 | 0.03 (0.02–0.05) | 0.22±0.10 | 0.22 (0.15–0.29) | 0.04±0.08 | 0 (0–0.13) | 0.15 |
| 8 | 0±0.01 | 0 (0–0.01) | 0.10±0.05 | 0.10 (0.06–0.14) | 0±0 | 0 (0–0) | 0.02±0.03 | 0 (0–0.05) | 0.154 |
| 9 | 0.09±0.16 | 0 (0–0.28) | 0.01±0.02 | 0.01 (0–0.02) | 0.03±0.05 | 0.03 (0–0.07) | 0.01±0.02 | 0 (0–0.03) | 0.954 |
| p2 | 0.745 |  | 0.527 |  | 0.259 |  | 0.920 |  |  |

**p1**: Intra-station seasonal variation (Friedman Test) **p2**: Inter-station seasonal differences (Kruskal-Wallis H Test) *p<0.05; **p<0.01

Table 9. Seasonal Variation of Mg Concentrations (µg/L, Mean ± SD)

| Station | Fall Mean ± SD | Fall Median (Min.–Max.) | Winter Mean ± SD | Winter Median (Min.–Max.) | Spring Mean ± SD | Spring Median (Min.–Max.) | Summer Mean ± SD | Summer Median (Min.–Max.) | p1 |
| --- | --- | --- | --- | --- | --- | --- | --- | --- | --- |
| 1 | 6384.58±1904.16 | 6320.58 (4513.22–8319.93) | 2730.53±1544.38 | 3209.98 (1003.29–3978.31) | 2469.81±51.30 | 2489 (2411.68–2508.74) | 3751.43±788.44 | 3817.80 (2931.91–4504.59) | 0.072 |
| 2 | 4780.56±237.22 | 4792.05 (4537.80–5011.82) | 3394.64±1670.55 | 3542.48 (1655.08–4986.36) | 3035.30±367.87 | 3244.78 (2610.54–3250.58) | 3974.28±563.38 | 4191.33 (3334.65–4396.86) | 0.172 |
| 3 | 5144.49±601.99 | 5057.71 (4590.60–5785.16) | 4631.03±381.34 | 4631.03 (4361.38–4900.68) | 3019.15±951.73 | 3228.94 (1980.02–3848.47) | 3847.77±443.59 | 4015.22 (3344.83–4183.26) | 0.145 |
| 4 | 4812.40±349.93 | 4970.40 (4411.33–5055.47) | 4905.05±111.11 | 4905.05 (4826.48–4983.62) | 3095.08±143.60 | 3169.68 (2930.31–3187.46) | 3501.43±568.54 | 3351.36 (3022.98–4129.96) | 0.145 |
| 5 | 5799.05±267.64 | 5803.83 (5529.04–6064.26) | 2993.61±487.39 | 2993.61 (2648.97–3338.25) | 3557.07±188.88 | 3557.07 (3423.51–3690.63) | 4800.30±762.59 | 5127.80 (3928.67–5344.44) | 0.112 |
| 6 | 5002.09±469.73 | 5191.00 (4467.32–5347.97) | 3552.42±51.77 | 3552.42 (3515.81–3589.02) | 4268.54±132.24 | 4268.54 (4175.03–4362.04) | 5047.90±613.08 | 5400.36 (4339.98–5403.36) | 0.241 |
| 7 | 6125.63±379.34 | 6147.29 (5735.92–6493.67) | 4517.63±103.61 | 4517.63 (4444.37–4590.89) | 4715.28±25.97 | 4715.28 (4696.91–4733.64) | 5698.89±691.35 | 6023.52 (4904.97–6168.17) | 0.308 |
| 8 | 6599.14±225.35 | 6530.70 (6415.95–6850.78) | 5611.80±76.35 | 5611.80 (5557.82–5665.79) | 4525.31±422.07 | 4525.31 (4226.87–4823.76) | 5863.98±445.78 | 6097.92 (5349.93–6144.09) | 0.145 |
| 9 | 5828.14±1003.85 | 6285.97 (4676.99–6521.46) | 5394.29±689.32 | 5394.29 (4906.87–5881.71) | 4952.40±631.72 | 4952.40 (4505.71–5399.10) | 5794.94±606.18 | 6132.46 (5095.13–6157.21) | 0.494 |
| p2 | 0.072 |  | 0.085 |  | 0.023* | 6, 7, 8, 9 > 1 | 0.016* | 7, 8, 9 > 1, 2 | |

**p1**: Intra-station seasonal variation (Friedman Test) **p2**: Inter-station seasonal differences (Kruskal-Wallis H Test) *p<0.05; **p<0.01

Table 10. Seasonal Variation of Mn Concentrations (µg/L, Mean ± SD)

| Station | Fall Mean ± SD | Fall Median (Min.–Max.) | Winter Mean ± SD | Winter Median (Min.–Max.) | Spring Mean ± SD | Spring Median (Min.–Max.) | Summer Mean ± SD | Summer Median (Min.–Max.) | p1 |
| --- | --- | --- | --- | --- | --- | --- | --- | --- | --- |
| 1 | 40.51±35.41 | 55.90 (0–65.62) | 12.05±6.07 | 9.55 (7.63–18.98) | 2.05±0.21 | 1.93 (1.92–2.29) | 15.34±10.54 | 12.86 (6.26–26.90) | 0.334 |
| 2 | 31.93±38.06 | 16.67 (3.86–75.26) | 36.14±21.51 | 33.71 (15.94–58.75) | 45.51±31.13 | 33.62 (22.08–80.84) | 10.63±2.60 | 10.48 (8.11–13.31) | 0.241 |
| 3 | 20.29±12.53 | 19.27 (8.30–33.29) | 1.39±1.26 | 1.39 (0.50–2.28) | 21.21±9.94 | 17.58 (13.60–32.46) | 22.92±27.46 | 8.83 (5.37–54.56) | 0.308 |
| 4 | 10.43±9.29 | 13.50 (0–17.80) | 3.52±2.39 | 3.52 (1.83–5.21) | 14.39±3.44 | 12.89 (11.94–18.33) | 12.53±12.07 | 9.78 (2.07–25.73) | 0.241 |
| 5 | 1.66±2.87 | 0 (0–4.97) | 1.79±0.71 | 1.79 (1.29–2.30) | 3.67±1.51 | 3.67 (2.60–4.74) | 2.92±1.09 | 3.40 (1.68–3.70) | 0.615 |
| 6 | 0.23±0.40 | 0 (0–0.70) | 3.98±3.14 | 3.98 (1.77–6.20) | 2.61±1.57 | 2.61 (1.50–3.73) | 1.73±0.77 | 1.37 (1.20–2.61) | 0.241 |
| 7 | 0.14±0.25 | 0 (0–0.43) | 0.63±0.08 | 0.63 (0.57–0.69) | 2.26±0.52 | 2.26 (1.89–2.63) | 0.89±0.34 | 1.05 (0.50–1.13) | 0.112 |
| 8 | 0.06±0.10 | 0 (0–0.17) | 0.40±0.48 | 0.40 (0.06–0.74) | 0.19±0.26 | 0.19 (0–0.37) | 0.16±0.10 | 0.14 (0.07–0.26) | 0.753 |
| 9 | 0.18±0.11 | 0.17 (0.07–0.30) | 0.07±0.09 | 0.07 (0–0.13) | 0.47±0.15 | 0.47 (0.36–0.57) | 0.63±0.57 | 0.51 (0.12–1.25) | 0.494 |
| p2 | 0.105 |  | 0.031* | 1, 2 > 7, 8, 9 | 0.013* | 2, 3, 4 > 8, 9 | 0.004** | 1, 2, 3, 4 > 7, 8, 9 | |

**p1**: Intra-station seasonal variation (Friedman Test) **p2**: Inter-station seasonal differences (Kruskal-Wallis H Test) *p<0.05; **p<0.01

Table 11. Seasonal Variation of Ni Concentrations (µg/L, Mean ± SD)

| Station | Fall Mean ± SD | Fall Median (Min.–Max.) | Winter Mean ± SD | Winter Median (Min.–Max.) | Spring Mean ± SD | Spring Median (Min.–Max.) | Summer Mean ± SD | Summer Median (Min.–Max.) | p1 |
| --- | --- | --- | --- | --- | --- | --- | --- | --- | --- |
| 1 | 0.53±0.92 | 0 (0–1.60) | 1.26±1.15 | 0.60 (0.59–2.59) | 0.11±0.15 | 0.04 (0–0.28) | 0.82±0.72 | 1.14 (0–1.33) | 0.183 |
| 2 | 0.15±0.13 | 0.20 (0–0.26) | 0.57±0.34 | 0.48 (0.28–0.95) | 1.38±2.39 | 0 (0–4.15) | 0.05±0.09 | 0 (0–0.16) | 0.254 |
| 3 | 0.05±0.08 | 0 (0–0.15) | 2.23±2.91 | 2.23 (0.17–4.29) | 3.39±4.47 | 0.82 (0.81–8.56) | 0.14±0.24 | 0 (0–0.42) | 0.12 |
| 4 | 0.45±0.48 | 0.40 (0–0.96) | 1.16±0.81 | 1.16 (0.58–1.74) | 0.24±0.21 | 0.32 (0–0.39) | 0.74±1.29 | 0 (0–2.23) | 0.5 |
| 5 | 2.23±1.82 | 3.20 (0.13–3.36) | 4.06±5.22 | 4.06 (0.37–7.75) | 0.35±0.38 | 0.35 (0.08–0.62) | 0.78±0.94 | 0.51 (0–1.82) | 0.494 |
| 6 | 0.76±1.07 | 0.29 (0–1.97) | 2.62±2.63 | 2.62 (0.76–4.48) | 0.31±0.43 | 0.31 (0–0.61) | 0.02±0.03 | 0 (0–0.06) | 0.145 |
| 7 | 0.45±0.78 | 0 (0–1.35) | 0.35±0.02 | 0.35 (0.34–0.37) | 0.45±0.49 | 0.45 (0.10–0.79) | 0.35±0.60 | 0 (0–1.04) | 0.984 |
| 8 | 0.68±1.18 | 0 (0–2.04) | 2.75±3.11 | 2.75 (0.55–4.95) | 0±0 | 0 (0–0) | 0±0 | 0 (0–0) | 0.112 |
| 9 | 1.09±1.51 | 0.47 (0–2.82) | 0.87±0.95 | 0.87 (0.20–1.55) | 2.86±2.95 | 2.86 (0.78–4.95) | 0.08±0.14 | 0 (0–0.25) | 0.241 |
| p2 | 0.632 |  | 0.709 |  | 0.141 |  | 0.686 |  |  |

**p1**: Intra-station seasonal variation (Friedman Test) **p2**: Inter-station seasonal differences (Kruskal-Wallis H Test) *p<0.05; **p<0.01

Table 12. Seasonal Variation of K Concentrations (µg/L, Mean ± SD)

| Station | Fall Mean ± SD | Fall Median (Min.–Max.) | Winter Mean ± SD | Winter Median (Min.–Max.) | Spring Mean ± SD | Spring Median (Min.–Max.) | Summer Mean ± SD | Summer Median (Min.–Max.) | p1 |
| --- | --- | --- | --- | --- | --- | --- | --- | --- | --- |
| 1 | 746.4±697.16 | 583.58 (145.05–1510.57) | 723.82±595.61 | 799.12 (94.14–1278.20) | 356.3±63.17 | 387.96 (283.56–397.38) | 661.69±320.26 | 699.91 (324.03–961.13) | 0.896 |
| 2 | 1026.29±305.72 | 1095.37 (691.94–1291.55) | 723.99±542.37 | 847.79 (130.42–1193.75) | 650.06±62.34 | 665.03 (581.59–703.55) | 697.91±41.33 | 694.03 (658.65–741.04) | 0.172 |
| 3 | 1085.57±350.47 | 1152.19 (706.57–1397.94) | 1211.55±265.89 | 1211.55 (1023.53–1399.56) | 635.81±127.11 | 652.84 (501.05–753.56) | 674.14±33.4 | 656.28 (653.47–712.67) | 0.187 |
| 4 | 1019.57±254.52 | 1115.37 (731.04–1212.29) | 1163.24±47.28 | 1163.24 (1129.81–1196.67) | 692.12±31.42 | 691.42 (661.05–723.88) | 643.11±136.23 | 676.15 (493.4–759.78) | 0.187 |
| 5 | 529.14±76.71 | 494.83 (475.58–617.02) | 633.77±44.3 | 633.77 (602.45–665.10) | 558.8±46 | 558.8 (526.27–591.32) | 609.44±100.75 | 619.79 (503.92–704.62) | 0.187 |
| 6 | 502.53±75.47 | 534.21 (416.38–556.99) | 680±207.86 | 680 (533.02–826.99) | 440.64±79.42 | 440.64 (384.48–496.80) | 504.9±43.27 | 507.86 (460.23–546.63) | 0.241 |
| 7 | 547.45±33.79 | 539.72 (518.19–584.43) | 543.29±92.35 | 543.29 (477.98–608.59) | 394.02±48.63 | 394.02 (359.63–428.41) | 518.22±53.53 | 517.8 (464.9–571.95) | 0.241 |
| 8 | 503.31±26.15 | 499.06 (479.55–531.32) | 477.39±56.68 | 477.39 (437.31–517.47) | 423.33±7.72 | 423.33 (417.87–428.79) | 515.2±84.73 | 484.71 (449.94–610.96) | 0.241 |
| 9 | 811.95±531.94 | 522.77 (487.24–1425.84) | 498.07±80.71 | 498.07 (441–555.15) | 427.07±48.53 | 427.07 (392.75–461.39) | 534±102.75 | 487.82 (462.45–651.74) | 0.494 |
| p2 | 0.129 |  | 0.343 |  | 0.027* | 2, 3, 4 > 1 | 0.186 |  |  |

**p1**: Intra-station seasonal variation (Friedman Test) **p2**: Inter-station seasonal differences (Kruskal-Wallis H Test) *p<0.05; **p<0.01
